# Hsa_circRNA_0001971 contributes to oral squamous cell carcinoma progression via miR‐186‐5p/Fibronectin type III domain containing 3B axis

**DOI:** 10.1002/jcla.24245

**Published:** 2022-01-21

**Authors:** Jiehua Zhang, Youjian Peng, Shengjun Jiang, Jun Li

**Affiliations:** ^1^ 117921 Department of Stomatology Renmin Hospital of Wuhan University Wuhan China; ^2^ Hubei Province Key Laboratory of Oral and Maxillofacial Development and Regeneration Wuhan China

**Keywords:** circRNA_0001971, FNDC3B, miR‐186‐5p, oral squamous cell carcinoma

## Abstract

**Background:**

Circular RNAs (circRNAs) are closely associated with the progression of oral squamous cell carcinoma (OSCC). circRNA_0001971 has been proved to accelerate the OSCC development. Here, we aim to identify the new molecular mechanism of hsa_circRNA_0001971 (circRNA_0001971) in OSCC.

**Methods:**

The levels of circRNA_0001971, miR‐186‐5p, and fibronectin type III domain containing 3B (FNDC3B) in tissues and cells were verified by qRT‐PCR or Western blotting. The interaction between circRNA_0001971, miR‐186‐5p, and FNDC3B was identified by bioinformatics analysis, luciferase assay, and RIP assay. The effect of circRNA_0001971/miR‐186‐5p/FNDC3B axis on OSCC cell proliferation, migration, and invasion by cell functional experiments including CCK8, wound healing, and transwell assays.

**Results:**

Our study displayed that circRNA_0001971 and FNDC3B were elevated in OSCC, whereas miR‐186‐5p was declined in OSCC. Silencing circRNA_0001971 attenuated the malignancy of OSCC cells by suppressing proliferation, migration, and invasion. In OSCC cells, circRNA_0001971 sponged miR‐186‐5p to enhance FNDC3B. Due to the interaction between circRNA_0001971, miR‐186‐5p, and FNDC3B, FNDC3B overexpression relieved the negative function of silencing circRNA_0001971 in OSCC cells.

**Conclusion:**

Overall, our study discovered that circRNA_0001971 was a tumor promoter in OSCC progression by targeting miR‐186‐5p/FNDC3B axis.

## Graphical abstract

circRNA_0001971 contributes to oral squamous cell carcinoma progression via sponging miR‐186‐5p to upregulate FNDC3B.

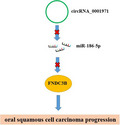

## 1 INTRODUCTION

Oral squamous cell carcinoma (OSCC) occurring in the epithelial tissue of oral mucosa accounts for 90% of all oral cancer cases worldwide.^1^, ^2^ It is worth noting that the OSCC prognosis is still poor though most of OSCC therapies including surgery, radiotherapy, and chemotherapy have been advanced.^3^, ^4^ High mortality rate such as lymph node metastasis or distant metastasis is responsibility for the poor prognosis of OSCC.^5^, ^6^ Therefore, the study on the molecular mechanisms underlying OSCC progression is worthy and important to identify novel therapeutic targets.

Circular RNAs (circRNAs) featured by covalently closed‐loop structures are considered as competitive endogenous RNAs (ceRNAs) of microRNA (miRNAs) to affect gene expression.^7^, ^8^ circRNAs previously regarded as accessory substance of splicing errors are proved to serve key roles in multiple human disease with the deep study on circRNA function.^9^, ^10^, ^11^, ^12^ During OSCC development, circRNAs are pointed out to act as key roles by ceRNA mechanism. For example, circIGHG contributed to OSCC progression and induced epithelial‐to‐mesenchymal transition via sponging miR‐142‐5p to regulate IGF2BP3 expression.^13^ Another circRNA circ_100533 was proved to be a tumor suppressor in OSCC by sponging miR‐933/GNAS axis.^14^ Hsa_circ_0001971 (circ_0001971), a novel circRNA, was only found in OSCC to enhance the OSCC progression by sponging miR‐194/miR‐204.^15^ However, the regulatory mechanism of circRNAs is complex, which may involve multiple miRNAs to regulating different expressions of genes. Therefore, the molecular mechanism of circ_0001971 is still necessary to be deeply explored.

Our study was to probe the role of circ_0001971 in OSCC progression and provide a novel molecular axis of circ_0001971 regulating OSCC progression by the ceRNA mechanism. Our findings might provide novel evidence for circ_0001971 potential as a target of OSCC treatment.

## 2 MATERIALS AND METHODS

### 2.1 Clinical samples collection

Thirty‐four paired OSCC samples and adjacent normal samples were collected from the patients diagnosed with OSCC in our hospital between January 2020 and May 2021. Each participant in our study signed informed consent, and the ethics committee of our hospital approved our study. The inclusion criteria in our study were as follows (1): All patients were diagnosed with OSCC for the first time (2); all the patients had not received chemotherapy and radiation before surgery; and (3) all patients signed informed consent. The exclusion criteria in our study were as follows (1): The patients with OSCC were diagnosed with other diseases (2); the patients had received chemotherapy or radiotherapy. Table 1 showed the clinical characteristics of all participant.

**TABLE 1:** Clinical characteristics of OSCC patients

Characteristic	Number of patients (%)
Total	34 (100)
Age	
≤60	24 (71)
>60	10 (29)
Sex	
Female	12 (35)
Male	22 (65)
Tumor location	
Buccal mucosa	18 (53)
Alveolus	10 (29)
Tongue	3 (9)
Lip	2 (6)
Retromolar area	1 (3)
Differentiation	
Well	11 (32)
Moderate	20 (59)
Poor	3 (9)
Stage	
I–II	13 (38)
III–IV	21 (62)
Tumor thickness (cm)	
≤0.7	8 (23)
0.8–1.4	14 (42)
≥1.5	12 (35)
Occult metastasis	
Present	7 (21)
Absent	27 (79)

### 2.2 Cell culture and cell transfection

Human oral epithelial cell line (HOEC) was provided by Procell Life Science & Technology Co., Ltd. SCC‐4 and CAL‐27 were two OSCC cell lines from ATCC (USA). HSC‐3 was another OSCC cell lines from Sigma‐Aldrich. For cell culture, DMEM medium containing 10% FBS was used to culture all cells at 37°C and 5% CO_2_ in an incubator.

For cell transfection, two siRNAs targeting circRNA_0001971 (si‐circ1 and si‐circ2), negative control of siRNA (si‐NC), miR‐186‐5p mimic, mimic‐NC, and FNDC3B overexpression pcDNA 3.1 vectors (OE‐FNDC3B) were purchased from RiboBio. The empty vector was the negative control of OE‐FNDC3B. At 70% confluence, the cells (SCC‐4 and HSC‐3) were transfected with 50 nM vectors mentioned above.

### 2.3 qRT‐PCR

TRIzol Reagent (Cat#: 15596018, Invitrogen) was applied to isolate total RNA from tissues and cells. Then, 1.5 μg isolated RNA was reverse transcribed to cDNA by SuperScript Reverse Transcriptase (Cat#: 18064014, Invitrogen) followed by performing qRT‐PCR using SYBR Premix Ex Taq Kit (Takara). The primer sequences are listed in Table 2. The relative expression was analyzed by 2^−ΔΔCT^ method with GAPDH or U6 as internal references.

**TABLE 2:** Primer sequences for qRT‐PCR in the study

Name	Primer sequences
circ_0001971	Forward (5′‐3′): GTGGACCTCATCCTCAAAGGG
	Reverse (5′‐3′): AGCTGGTGACAGACAGGTTCT
miR‐186	Forward (5′‐3′): CGGCGGCAAAGAATTCTCCTT
	Reverse (5′‐3′): CAGTGCGTGTCGTGGAGT
FNDC3B	Forward (5′‐3′): GGGACAGACACCCGTTTTGA
	Reverse (5′‐3′): GTGTTGCCCACGGTAATGCT
GAPDH	Forward (5′‐3′): ATTCCACCCATGGCAAATTC
	Reverse (5′‐3′): TGGGATTTCCATTGATGA
U6	Forward (5′‐3′): CACCACGUUUAUACGCCGGUG
	Reverse (5′‐3′): CACCACGTTTATACGCCGGTG

### 2.4 The detection of cell proliferation, cell migration, and cell invasion

Proliferation of SCC‐4 and HSC‐3 cells was assessed via Cell Counting Kit 8 (CCK8, Dojindo). The transfected cells (5,000 cells/well) were cultured in 96‐well plates, and 10 μl/well CCK8 reagent was added at different times (0, 24, 48, and 72 h). The OD value was determined by a microplate reader at 450 nm.

Wound‐healing assay was carried out to identify the change in cell migration. The transfected cells (1 × 10^6^ cells/well) were cultured in 6‐well plates until >90% confluence followed by creating the artificial wounds with 200 μl pipette tips. After removing the exfoliated cells, the cells were continued to culture in serum‐free medium for 24 h. Finally, the images of wound healing at 0 and 24 h were photographed by a light microscope.

As for cell invasion, transwell assay was performed with the membranes coated with Matrigel (Millipore) for incubation overnight. The 3 × 10^4^ cells after transfection were added in the upper chamber without serum. For lower chamber, the medium containing 10% FBS was added. The membranes were stained after incubation cells for 48 h, and the images of invaded cells in the membranes were photographed by a light microscope.

### 2.5 Bioinformatics analysis

GSE82064 from GEO Datasets was a miRNA microarray storing the differentially expressed miRNAs in OSCC. By setting adj.*p* < 0.05 and log_2_FC < −1 as the screening criteria, the downregulated miRNAs in OSCC were identified. circInteractome,^16^ an online tool, was used to predict the miRNAs binding to circRNA_0001971, whereas TargetScan,^17^ an online tool, was used to predict the mRNAs binding to miR‐186‐5p. GEPIA^18^ storing the differentially expressed mRNAs in OSCC samples was used to identify the upregulated mRNAs with adj.*p* < 0.05 and log_2_FC > 1 as the screening criteria. Besides, the data from TCGA were used to show the mRNAs expression in OSCC samples and normal samples.

### 2.6 Luciferase assay

The binding sites of miR‐186‐5p for circRNA_0001971 and FNDC3B were predicted by circInteractome and TargetScan, respectively. According to the prediction, the wild‐type circRNA_0001971 (WT circRNA_0001971), wild‐type FNDC3B (WT FNDC3B), and mutant circRNA_0001971 (MUT circRNA_0001971) were designed and inserted into psiCHECK‐2 vector. Due to the two binding sites between miR‐186‐5p and FNDC3B, MUT1 FNDC3B (position: 2967–2974), MUT2 FNDC3B (position: 3212–3218), and co‐MUT FNDC3B (MUT1+MUT2) were designed and inserted into psiCHECK‐2 vector. Then, these vectors were transfected into OSCC cells together with miR‐186‐5p mimic/mimic‐NC. The luciferase activity was measured by Dual‐Luciferase Reporter Assay Kit (Cat#: DL101‐01, Vazyme) after 48 h transfection.

### 2.7 RIP assay

Magna RIP Kit (Millipore, USA) was purchased for performing RIP assay. The OSCC cells transfected with miR‐186‐5p mimic or mimic‐NC for 48 h were collected and lysed with RIP buffer. Then, the magnetic beads coated with anti‐Ago2 or anti‐IgG were added to cell lysates at 4°C overnight. Finally, the immunoprecipitated RNA was isolated to measure the enrichment of circRNA_0001971 by qRT‐PCR. The sequences of circRNA_0001971 primers are listed in Table 2.

### 2.8 Western blotting

For detecting the protein expression, RIPA lysis buffer (Beyotime, China) was first used to isolate the total proteins from cells. Then, the isolated proteins were separated by 10% SDS‐PAGE followed by transferring onto PVDF membranes (Beyotime, China). Next, the membranes after blocking with fat‐free milk were incubated with anti‐FNDC3B (Cat#: sc‐393997, Santa Cruz Biotechnology) or anti‐GAPDH (Cat#: sc‐365062, Santa Cruz Biotechnology) followed by incubating with anti‐mouse IgG‐HRP (Cat#: sc‐2005, Santa Cruz Biotechnology). Finally, the protein was visualized using Western Blotting Luminol Reagent (Cat#: sc‐2048, Santa Cruz Biotechnology) and UltraCruz Autoradiography Film (Cat#: sc‐201696, Santa Cruz Biotechnology).

### 2.9 Statistical analysis

All experimental values were analyzed as mean ± SD from three independent experiments. The differences for two groups were identified by paired student's *t* test, whereas ANOVA was applied to identify the differences for >2 groups. *p* value < 0.05 was statistically significant.

## 3 RESULTS

### 3.1 The role of circRNA_0001971 in OSCC cells

**FIGURE 1:**
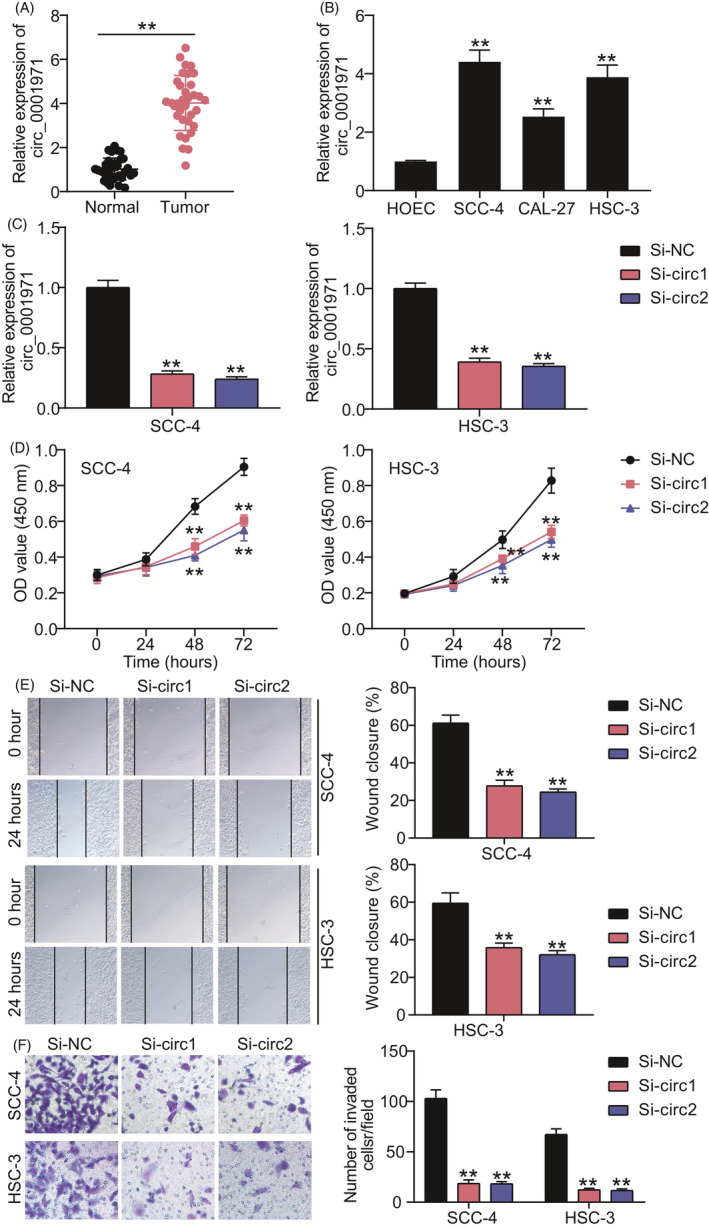
Role of circRNA_0001971 in OSCC cells. (A and B) The high expression of circRNA_0001971 in OSCC tissues (A) and OSCC cells (B) by qRT‐PCR. Normal, adjacent normal tissues. Tumor, OSCC tissues. HOEC, human oral epithelial cells. SCC‐4, CAL‐27, and HSC‐3, three OSCC cell lines. **p < 0.01 versus Normal or HOEC. (B) The expression of circRNA_0001971 in SCC‐4 and HSC‐3 cells decreased after transfection of si‐circ1 or si‐circ2. (D) circRNA_0001971 knockdown inhibited cell proliferation in SCC‐4 and HSC‐3 cells by CCK8 assay. (E) circRNA_0001971 knockdown impaired cell migration capability in SCC‐4 and HSC‐3 cells by wound healing assay. (F) circRNA_0001971 knockdown suppressed cell invasion in SCC‐4 and HSC‐3 cells by transwell assay. (C–F) si‐NC, the negative control of siRNA. Si‐circ1 and si‐circ2, two siRNAs of circRNA_0001971. **p < 0.01 versus.si‐NC

After performing qRT‐PCR, the level of circRNA_0001971 in OSCC tissues and cells was found to be upregulated compared with adjacent normal tissues or HOEC (Figure 1A, B). Since the expression of circRNA_0001971 increased by almost fourfold in SCC‐4 and HSC‐3 OSCC cells, the transfection efficiency of two siRNAs targeting circRNA_0001971 was determined in these cell lines (Figure 1C). Then, the CCK8 assay displayed that silencing circRNA_0001971 suppressed cell proliferation in both two OSCC cells (Figure 1D). The wound closure rate detected by wound‐healing assay was decreased by approximately 50% in silencing circRNA_0001971 group, suggesting that circRNA_0001971 knockdown inhibited cell migration in OSCC cells (Figure 1E). Moreover, the transwell assay discovered that silencing circRNA_0001971 impaired invasion ability of SCC‐4 and HSC‐3 cells (Figure 1F). Our data proved the inhibitory role of silencing circRNA_0001971 in OSCC cells.

### 3.2 miR‐186‐5p was sponged by circRNA_0001971 in OSCC cells

**FIGURE 2:**
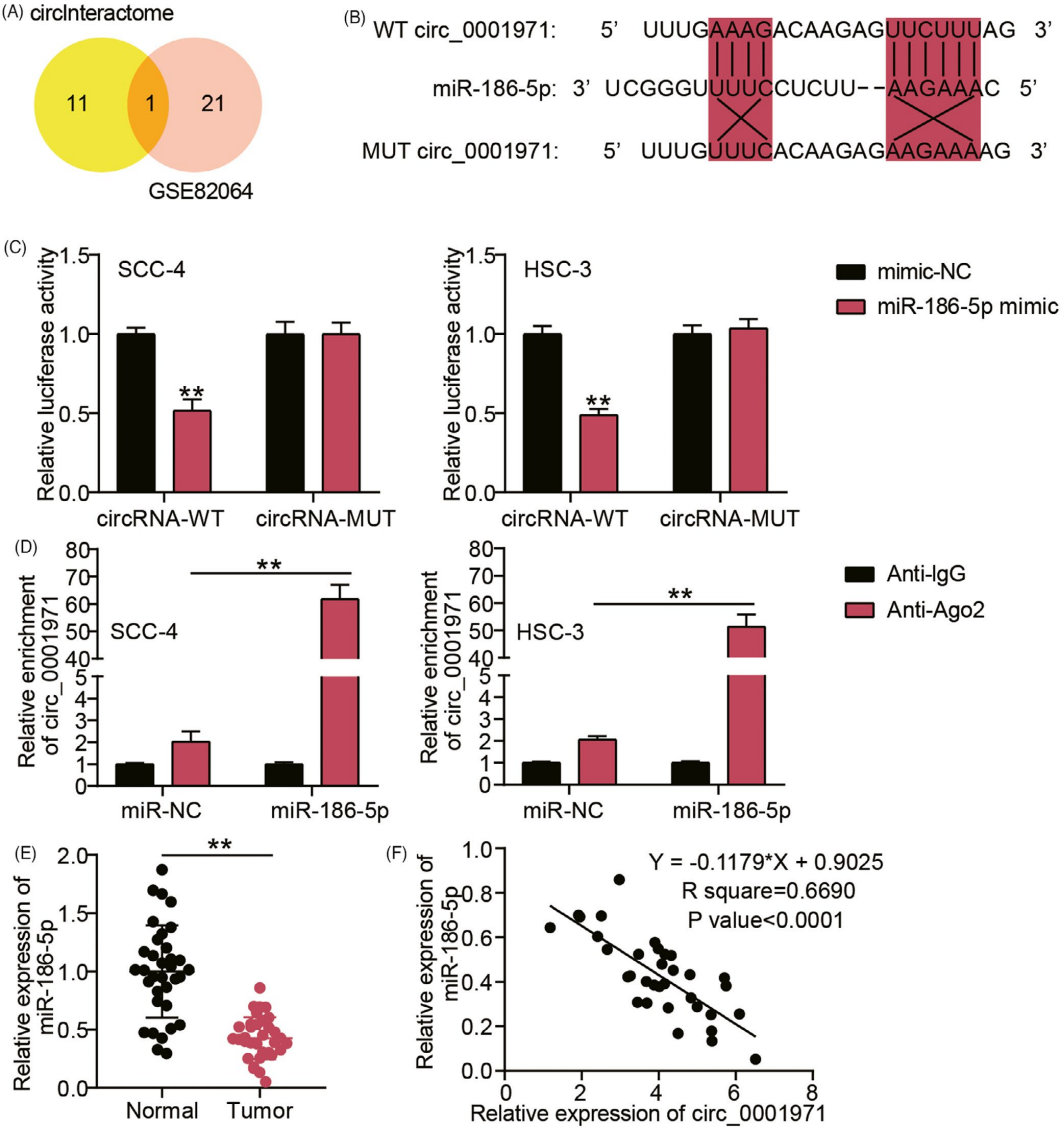
miR‐186‐5p was sponged by circRNA_0001971 in OSCC cells. (A) Only one miRNA (miR‐186‐5p) was overlapped from circInteractome and GSE82064. circInteractome, a tool to predict the downstream miRNAs of circRNA_0001971. GSE82064, a miRNA microarray to screen the downregulated miRNAs in OSCC samples with adjusted P (adj.P) < 0.05 and logFC < −1. (B) The sequences of wild‐type (WT) and mutant (MUT) circRNA_0001971. (C) The co‐transfection of WT circRNA_0001971 vectors and miR‐186‐5p mimic decreased the luciferase activity in SCC‐4 and HSC‐3 cells by luciferase assay. mimic‐NC, the negative control of mimic. **p < 0.01 versus co‐transfection of WT circRNA_0001971 vectors and mimic‐NC. (D) miR‐186‐5p could bind to circRNA_0001971 by RIP assay. **p < 0.01. (E) The low expression of miR‐186‐5p in OSCC tissues. Normal, adjacent normal tissues. Tumor, OSCC tissues by qRT‐PCR. **p < 0.01. (F) miR‐186‐5p expression was negatively correlated to circRNA_0001971 expression in OSCC samples by Pearson correlation analysis

circInteractome was an online tool to predict the downstream miRNAs of circRNA_0001971, as well as GSE82064 downloaded from GEO database aimed to screen the downregulated miRNAs in OSCC samples with adj.*p* < 0.05 and log_2_FC < −1. It was found that only one miRNA (miR‐186‐5p) was overlapped from circInteractome and GSE82064 (Figure 2A). According to the prediction by circInteractome, we designed the WT and MUT circRNA_0001971 vectors, and the sequences of WT and MUT circRNA_0001971 are shown in Figure 2B. The luciferase assay showed that co‐transfection of WT circRNA_0001971 vectors and miR‐186‐5p mimic reduced the luciferase activity (Figure 2C). The RIP assay further identified circRNA_0001971 as a sponge of miR‐186‐5p (Figure 2D). In our clinical samples, miR‐186‐5p expression decreased by 50% in OSCC samples (Figure 2E), as well as the level of miR‐186‐5p was negatively correlated to that of circRNA_0001971 in OSCC samples (Figure 2F). These findings suggested that circRNA_0001971 could sponge miR‐186‐5p in OSCC cells.

### 3.3 FNDC3B was a target of miR‐186‐5p in OSCC cells

**FIGURE 3:**
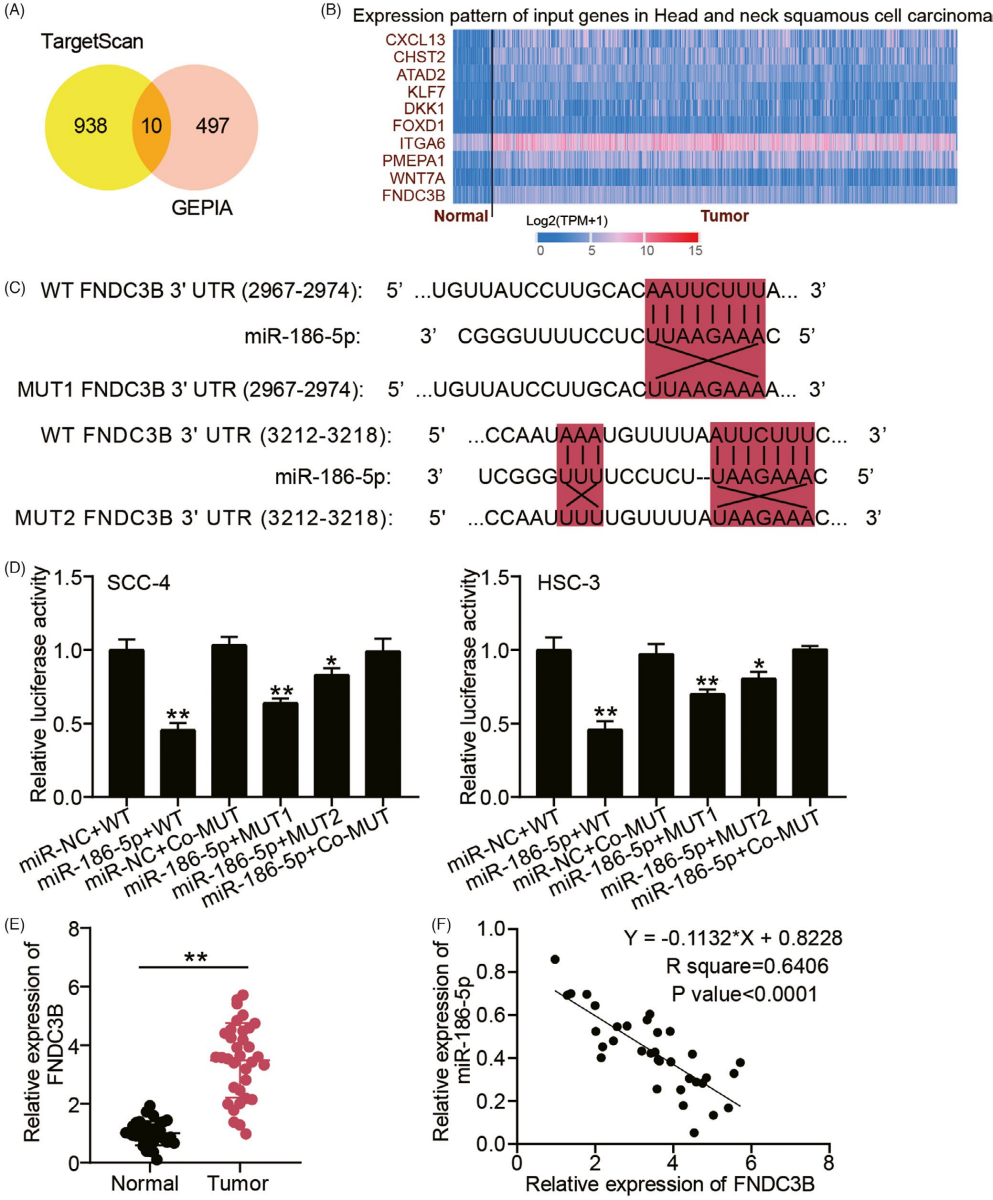
FNDC3B was a target gene of miR‐186‐5p in OSCC cells. (A) Ten mRNAs were overlapped from TargetScan and GEPIA. TargetScan, an online tool to predict the target genes for miR‐186‐5p. GEPIA, an online tool to screen out the upregulated mRNAs in OSCC samples with adj.*p *< 0.01 and logFC > 1. (B) The expression of ten mRNAs in head and neck squamous cell carcinoma according to the data from TCGA. (C) The sequences of WT and MUT FNDC3B vectors. (D) The co‐transfection of miR‐186‐5p mimic and WT/MUT1/MUT2 FNDC3B vectors reduced the luciferase activity by luciferase assay. (E) The high expression of FNDC3B in OSCC tissues by qRT‐PCR. Normal, adjacent normal tissues. Tumor, OSCC tissues by qRT‐PCR. **p < 0.01. (F) FNDC3B expression was negatively correlated to miR‐186‐5p expression in OSCC tissues by Pearson correlation analysis

To further explore the targets of miR‐186‐5p, TargetScan was used to predict the target genes for miR‐186‐5p, as well as GEPIA, an online tool, was applied to verify the upregulated mRNAs in OSCC with adj.*p* < 0.01 and log_2_FC > 1. As shown in Figure 3A, ten mRNAs were overlapped from TargetScan and GEPIA. According to the data from TCGA, FNDC3B was upregulated in head and neck squamous cell carcinoma (Figure 3B) and was found to be an oncogene in tongue squamous cell carcinoma.^19^ Therefore, FNDC3B was identified as the interested gene. The sequences of WT and MUT FNDC3B vectors are displayed in Figure 3C. After performing luciferase assay, the luciferase activity reduced the most in co‐transfection of miR‐186‐5p mimic and WT FNDC3B group, and MUT1 or MUT2 FNDC3B together with miR‐186‐5p mimic group induced a slight decrease in luciferase activity (Figure 3D). By qRT‐PCR, FNDC3B was found to be upregulated by 3.5‐fold in OSCC samples (Figure 3E), and its level was negatively correlated to miR‐186‐5p level in OSCC samples (Figure 3F). All the data proved that FNDC3B was a target gene of miR‐186‐5p.

### 3.4 FNDC3B overexpression partly abrogated the effect of circRNA_0001971 knockdown on OSCC cells

**FIGURE 4:**
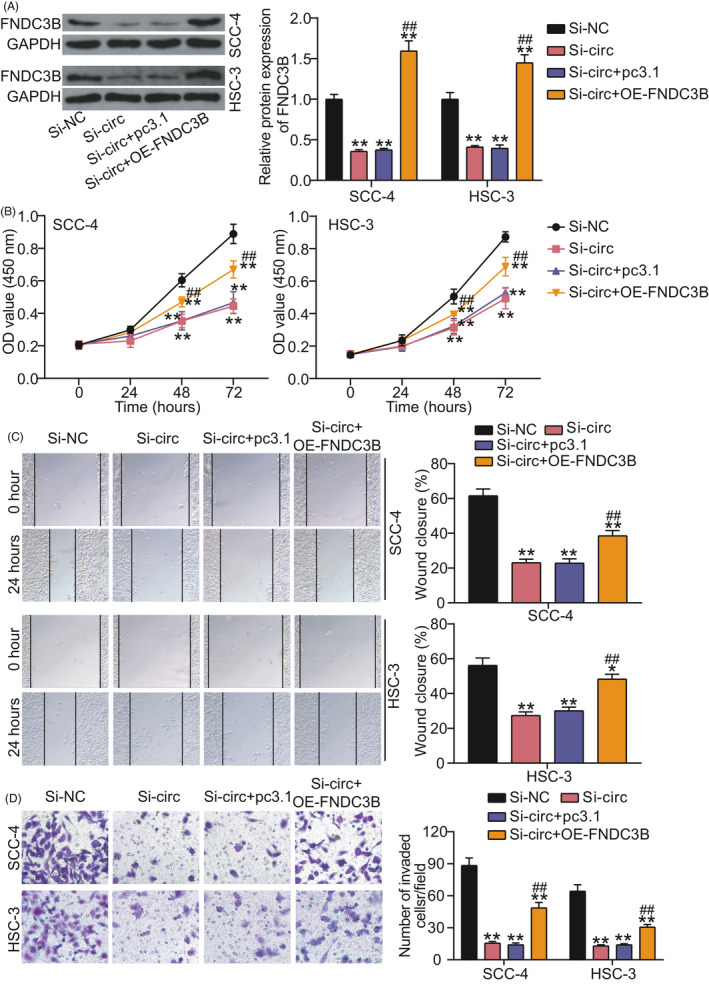
FNDC3B overexpression partly abrogated the effect of circRNA_0001971 knockdown on OSCC cells. (A) The expression of FNDC3B protein in SCC‐4 and HSC‐3 cells by Western blotting. (B) FNDC3B overexpression relieved the inhibitory effect of circRNA_0001971 knockdown on cell proliferation. (C) FNDC3B overexpression abrogated the inhibitory effect of circRNA_0001971 knockdown on cell migration. (D) FNDC3B overexpression attenuated the inhibitory effect of circRNA_0001971 knockdown on cell invasion. si‐NC, the negative control of siRNA. Si‐circ, siRNA of circRNA_0001971. OE‐FNDC3B, FNDC3B overexpression. **p < 0.01 versus si‐NC. ^##^ *p *< 0.01 versus si‐circ

The result from Western blotting proved that circRNA_0001971 knockdown induced around 40% downregulation of FNDC3B, whereas FNDC3B overexpression vectors overturned the effect of circRNA_0001971 knockdown on FNDC3B level in OSCC cells (Figure 4A). By CCK8 assay, FNDC3B overexpression was proved to partly relieve the decrease in cell proliferation induced by circRNA_0001971 knockdown in OSCC cells (Figure 4B). The wound‐healing assay and transwell assay discovered that the declined cell migration (Figure 4C) and invasion (Figure 4D) caused by circRNA_0001971 knockdown could be partly abrogated by co‐transfecting with FNDC3B overexpression vectors. Our results revealed that the upregulation of FNDC3B partly attenuated the negative effect of circRNA_0001971 knockdown on OSCC cell malignancy.

## 4 DISCUSSION

circRNAs have been proved to act as the key roles in OSCC development. Here, we found that a novel circRNA (circRNA_0001971) was upregulated in OSCC, and silencing circRNA_0001971 inhibited proliferation, migration, and invasion of OSCC cells. Meanwhile, circRNA_0001971 could upregulate FNDC3B by sponging miR‐186‐5p in OSCC cells. Besides, our study also proved that the effect of silencing circRNA_0001971 on OSCC cells was attenuated by FNDC3B overexpression due to the interaction between circRNA_0001971, miR‐186‐5p, and FNDC3B.

Increasing studies reveal that the abnormal expression of circRNAs is closely associated with OSCC progression; hence, circRNAs are considered as the key biomarkers of OSCC.^14^, ^20^, ^21^, ^22^ For instance, circ_0008309 with low expression was proved to be correlated with pathological differentiation, and circ_0008309 upregulated ATXN1 expression by sponging miR‐136‐5p and miR‐382‐5p in OSCC cells.^22^ circ‐PVT1 functioning as a miR‐106a‐5p sponge promoted OSCC cell growth and metastasis by upregulating HK2.^23^ Our study revealed that circRNA_0001971 was elevated in OSCC, and silencing circRNA_0001971 suppressed the malignancy of OSCC cells, which was consistent with the conclusion from X Tan et al.^15^ Nevertheless, X Tan et al. found circRNA_0001971 could sponge miR‐194 and miR‐204 in OSCC cells. Here, we found that circRNA_0001971 was a miR‐186‐5p sponge to upregulate FNDC3B in OSCC cells, which was different from X Tan et al.

microRNAs (miRNAs) have been reported as the downstream of circRNAs according to the ceRNA mechanism.^7^, ^24^ Here, we found miR‐186‐5p was a downstream of circRNA_0001971 in OSCC cells by bioinformatics analysis, luciferase assay, and RIP assay. miR‐186‐5p, a member of miRNAs, has been studied in human cancer, and its anticancer role is proved in various cancer types. For example, the anticancer roles of miR‐186‐5p were observed in gastric cancer,^25^ colorectal cancer,^26^ and ovarian cancer.^27^ In OSCC, multiple studies found miR‐186 was a tumor suppressor in OSCC by targeting FUT8^28^ and SHP2.^29^ Here, we for the first time proved that miR‐186‐5p was proved to be downregulated in OSCC samples, and FNDC3B was a target of miR‐186‐5p in OSCC cells.

Fibronectin type III domain containing 3B (FNDC3B) has been identified as a modulator of osteoblast and adipocyte differentiation.^19^ In recent years, many evidence proved the positive role of FNDC3B in colorectal cancer,^30^ cervical cancer,^31^ glioblastoma,^32^ and tongue squamous cell carcinoma cells.^19^ However, FNDC3B has not been explored in OSCC. In this study, FNDC3B with high expression was observed in OSCC samples, and its overexpression partly overturned the effect of circRNA_0001971 on OSCC progression in vitro owing to FNDC3B as the downstream of circRNA_0001971/miR‐186‐5p.

Our study proved the role of circRNA_0001971/miR‐186‐5p/FNDC3B axis in OSCC, but there are some limitations in this study. First of all, all the experiments were performed in clinical samples or cell samples, and the lack of experiments in vivo was limited the extension of our conclusion. Then, the clinical samples were collected in recent years so that we could analyze the relationship between circRNA_0001971/miR‐186‐5p/FNDC3B axis and prognosis of OSCC. Besides, the key signaling pathway of FNDC3B should be further explored in OSCC.

In summary, our study discovered that circRNA_0001971 was upregulated in OSCC and accelerated the progression of OSCC in vitro by ceRNA mechanism to sponge miR‐186‐5p/FNDC3B axis. Our conclusion might provide novel evidence for circ_0001971 potential as a target of OSCC therapy.

## CONFLICT OF INTEREST

The authors have no conflict of interests.

## AUTHOR CONTRIBUTIONS

JHZ designed this study, performed the experiments, and drafted the article. YJP analyzed the data and provided the funding. SJJ collected materials and resources. JL reviewed and edited the article.

## INFORMED CONSENT

Informed consent was obtained from all patients.

## Data Availability

All data and materials in the current study are available from the corresponding author if request is reasonable.
